# A Numerical Study of Scalable Cardiac Electro-Mechanical Solvers on HPC Architectures

**DOI:** 10.3389/fphys.2018.00268

**Published:** 2018-04-05

**Authors:** Piero Colli Franzone, Luca F. Pavarino, Simone Scacchi

**Affiliations:** ^1^Department of Mathematics, University of Pavia, Pavia, Italy; ^2^Department of Mathematics, University of Milano, Milan, Italy

**Keywords:** domain decomposition preconditioners, cardiac electro-mechanics, bidomain model, scalable parallel solvers, re-entrant waves, mechano-electric feedback

## Abstract

We introduce and study some scalable domain decomposition preconditioners for cardiac electro-mechanical 3D simulations on parallel HPC (High Performance Computing) architectures. The electro-mechanical model of the cardiac tissue is composed of four coupled sub-models: (1) the static finite elasticity equations for the transversely isotropic deformation of the cardiac tissue; (2) the active tension model describing the dynamics of the intracellular calcium, cross-bridge binding and myofilament tension; (3) the anisotropic Bidomain model describing the evolution of the intra- and extra-cellular potentials in the deforming cardiac tissue; and (4) the ionic membrane model describing the dynamics of ionic currents, gating variables, ionic concentrations and stretch-activated channels. This strongly coupled electro-mechanical model is discretized in time with a splitting semi-implicit technique and in space with isoparametric finite elements. The resulting scalable parallel solver is based on Multilevel Additive Schwarz preconditioners for the solution of the Bidomain system and on BDDC preconditioned Newton-Krylov solvers for the non-linear finite elasticity system. The results of several 3D parallel simulations show the scalability of both linear and non-linear solvers and their application to the study of both physiological excitation-contraction cardiac dynamics and re-entrant waves in the presence of different mechano-electrical feedbacks.

## 1. Introduction

In recent years, several areas of medicine, and in particular cardiology, have undergone a cultural revolution generated by new findings that have emerged from molecular biology. This new knowledge has helped to identify, for each disease and for each patient, the specific mechanisms of the disease and the resulting medical treatments, leading to the so-called personalized medicine. For example, the use of mathematical models with parameters for the individual patient-specific characteristics could allow cardiologists to predict the effectiveness of anti-arrhythmic drug treatments or the proper installation of implantable defibrillators (see e.g., Nordsletten et al., 2011; Constantino et al., 2012; Lamata et al., 2015; Trayanova and Chang, 2016).

The spatio-temporal evolution of the electrical impulse in the cardiac tissue and the subsequent process of cardiac contraction-relaxation are quantitatively described by the cardiac electro-mechanical coupling model, which consists of the following four sub-models:

- the **static finite elasticity model** describing the deformation of cardiac tissue, derived from an anisotropic strain energy function which characterizes the passive mechanical properties of the myocardium;
- the **active tension system** of non-linear ordinary differential equations (ODEs), describing the dynamics of the intracellular calcium, cross-bridge binding and myofilament tension;
- the **anisotropic Bidomain model** of the cardiac tissue, which is a non-linear system of two partial differential equations (PDEs) of reaction-diffusion type, describing the spatio-temporal evolution of the intra- and extracellular electric potentials in the cardiac tissue;
- the **ionic membrane model** of the cardiac myocyte, a stiff system of ODEs describing the dynamics of ionic currents, gating variables, ionic concentrations and stretch-activated channels.

The theoretical and numerical challenges posed by this complex non-linear electro-mechanical model are very interesting. Indeed, the theoretical analysis of the well-posedness of the cardiac electro-mechanical coupling model is still an open problem, as well as the convergence analysis of its finite element approximation. On the numerical level, the very different space and time scales associated with the electrical and mechanical sub-models, as well as their non-linear and multiphysics interactions, make the approximation and simulation of the cardiac electro-mechanical coupling model a very demanding and expensive computational task.

In the last decade, several groups have performed cardiac computational studies based on three-dimensional electrical and electro-mechanical simulations (see Pathmanathan and Whiteley, 2009; Göktepe and Kuhl, 2010; Keldermann et al., 2010; Gurev et al., 2011; Trayanova et al., 2011; Land et al., 2012b; Nobile et al., 2012; Rossi et al., 2012; Dal et al., 2013; Sundnes et al., 2014; Favino et al., 2016). However, the computational costs required by the solution of the mathematical models describing the cardiac bioelectrical and mechanical activity are still too high to allow their use in a clinical setting. Therefore, there is a strong effort in the research community to develop effective computational tools and to speedup the simulation of the cardiac electro-mechanical activity (see e.g., Vázquez et al., 2011; Lafortune et al., 2012; Washio et al., 2013; Aguado-Sierra et al., 2015; Gurev et al., 2015; Land et al., 2015; Augustin et al., 2016).

Among the most efficient high-performance solvers for these complex cardiac models are parallel iterative methods, such as the Preconditioned Conjugate Gradient method (PCG) and Generalized Minimal Residual Method (GMRES), accelerated by proper scalable preconditioners. For the bioelectrical component modeled by the Bidomain system, several types of preconditioners have been proposed, such as Block Jacobi (BJ) preconditioners employing an incomplete LU factorization (ILU) for each block (Colli Franzone and Pavarino, 2004), other kinds of block preconditioners (Gerardo-Giorda et al., 2009; Chen et al., 2017). geometric multigrid (Sundnes et al., 2002; Weber dos Santos et al., 2004), algebraic multigrid (Plank et al., 2007; Pennacchio and Simoncini, 2009, 2011), and domain decomposition preconditioners such as Multilevel Schwarz (Pavarino and Scacchi, 2008; Scacchi, 2008, 2011; Munteanu et al., 2009; Pavarino and Scacchi, 2011; Charawi, 2017), Neumann-Neumann and BDDC (Zampini, 2013, 2014). For a general introduction to Domain Decomposition methods we refer the interested reader to the monograph (Toselli and Widlund, 2005). More recently, the study of efficient parallel solvers and preconditioners has been extended also to cardiac electro-mechanical models (see e.g., Colli Franzone et al., 2015; Gurev et al., 2015; Pavarino et al., 2015; Augustin et al., 2016; Colli Franzone et al., 2016a,b, 2017) and to cardiac and cardiovascular flow (see e.g., Quarteroni et al., 2017a,b).

The goal of this work is to study the performance of our parallel electro-mechanical solver in three-dimensional left-ventricular simulations on two different HPC (High Performance Computing) architectures. The finite element parallel solver we have developed is based on Multilevel Additive Schwarz preconditioners accelerated by PCG for solving the discretized Bidomain system and on Newton-Krylov methods with Balancing Domain Decomposition by Constraints (BDDC) preconditioners for solving the discretized non-linear finite elasticity system. Extensive numerical simulations have shown the scalability of both linear and non-linear solvers and their effectiveness in the study of the physiological excitation-contraction cardiac dynamics and of re-entrant waves in the presence of different mechano-electrical feedbacks.

The paper is organized as follows. The main four electro-mechanical cardiac sub-models are briefly introduced in section 2 and discretized in time and space in section 3, where the main computational kernels, parallel solvers and preconditioners are also described. Section 4 contains the main results of the paper obtained in large-scale 3D simulations using high-performance parallel architectures.

## 2. Electro-mechanical cardiac models

**Figure 1:**
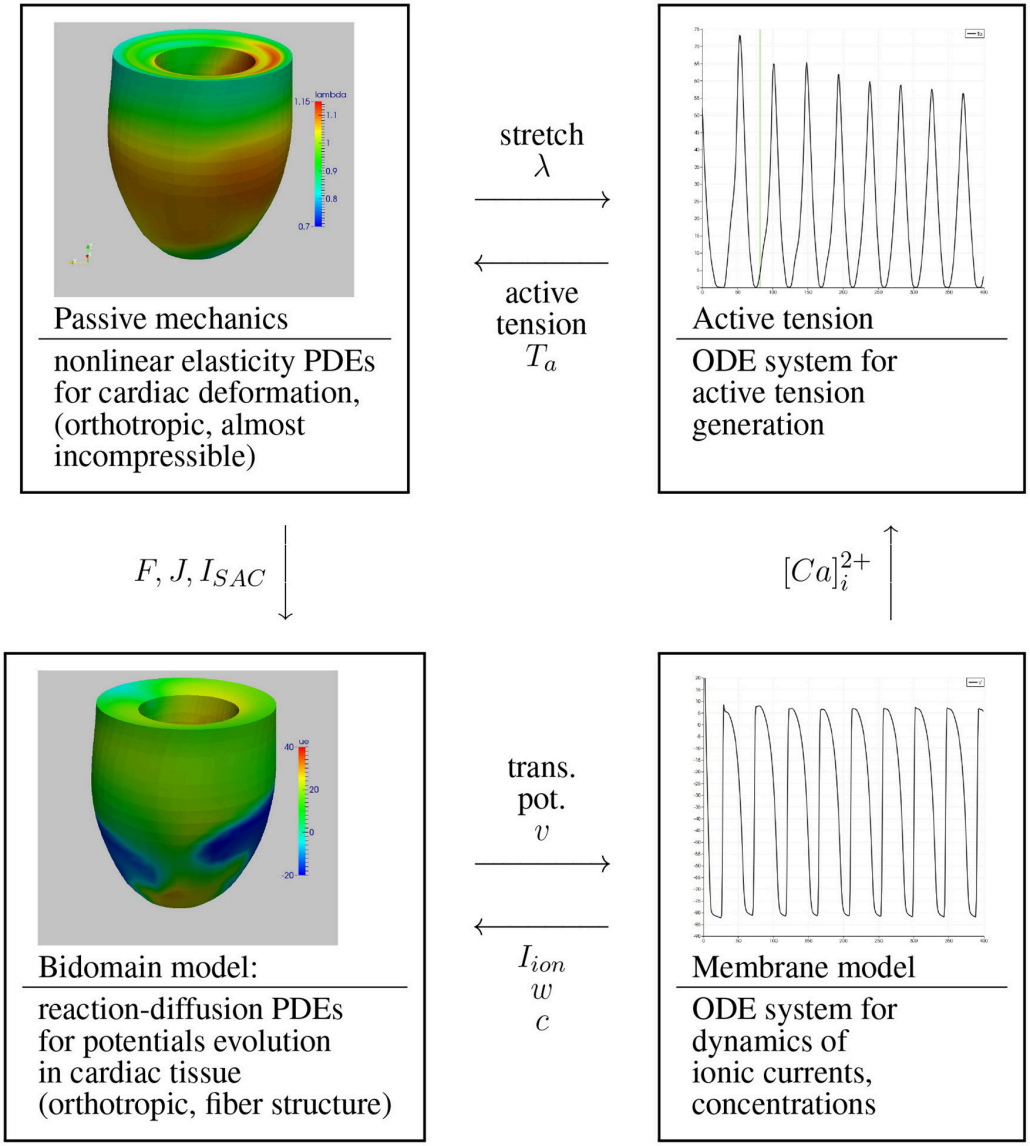
The four electro-mechanical submodels.

We conside a cardiac electro-mechanical coupling model consisting of the following four coupled sub-models; see also Figure 1.

### 2.1. Cardiac tissue mechanical model

We assume a quasi-steady state regime and model the cardiac tissue as a non-linear hyperelastic material satisfying the equilibrium equation

(1) $$\begin{array}{l} \mathrm{Div}\left(\text{F}\text{S}\right)=0,\;\text{X}\in \hat{\Omega }, \end{array}$$

with appropriate boundary conditions, where we denote by **x** = **x**(**X**, *t*) the spatial coordinates of the deformed cardiac domain Ω(*t*) at time *t*, by $\text{X}={\left(X_{1},X_{2},X_{3}\right)}^{T}$ the material coordinates of the undeformed cardiac domain $\hat{\Omega }$, by $\text{F}\left(\text{X},t\right)=\frac{\partial \text{x}}{\partial \text{X}}$ the deformation gradient and by **u**(**X**, *t*) = **x** − **X** the displacement field. Following the active stress approach, the second Piola-Kirchhoff stress tensor **S** is written as the sum of passive (*pas*), volumetric (*vol*) and active (*act*) components, i.e.,

(2) $$\begin{array}{l} \text{S}={\text{S}}^{pas}+{\text{S}}^{vol}+{\text{S}}^{act}. \end{array}$$

The passive and volumetric terms of **S** are defined as

$$\begin{array}{l} S_{ij}^{pas,vol}=\frac{1}{2}\left(\frac{\partial W^{pas,vol}}{\partial E_{ij}}+\frac{\partial W^{pas,vol}}{\partial E_{ji}}\right)\;i,j=1,2,3, \end{array}$$

where $\text{E}=\frac{1}{2}\left(\text{C}-\text{I}\right)$ is the Green-Lagrange strain tensor and *W*^*pas*^ is an exponential strain energy function describing the myocardium as an hyperelastic material transversely isotropic (derived form the orthotropic law proposed in Holzapfel and Ogden, 2009; Eriksson et al., 2013)

(3) $$\begin{array}{l} W^{pas}=\frac{a}{2b}\left(e^{b\left(I_{1}-3\right)}-1\right)+\underset{i=l,n}{\sum }\frac{a_{i}}{2b_{i}}\left(e^{b_{i}{\left(I_{4i}-1\right)}^{2}}-1\right)\\ +\;\frac{a_{ln}}{2b_{ln}}\left(e^{b_{ln}I_{8ln}^{2}}-1\right), \end{array}$$

where *a, b, a*_(*l,n,ln*)_, *b*_(*l,n,ln*)_ are positive material parameters and

$$\begin{array}{l} I_{4l}={\hat{\text{a}}}_{l}^{T}\text{C}{\hat{\text{a}}}_{l},\;I_{4n}={\hat{\text{a}}}_{n}^{T}\text{C}{\hat{\text{a}}}_{n},\;I_{8ln}={\hat{\text{a}}}_{l}^{T}\text{C}{\hat{\text{a}}}_{n}. \end{array}$$

We did not employ an isochoric-deviatoric decomposition of the deformation gradient tensor. The volumetric term *W*^*vol*^ = *K*(*J* − 1)^2^ is a penalization term added to enforce the nearly incompressibility of the myocardium, where *K* is a positive bulk modulus and *J* = *det***F**. The model is closed by imposing boundary conditions of mixed Dirichlet and traction type.

### 2.2. Mechanical active tension model

The active tension generation model is based on calcium kinetic and myofilament dynamics. Here we consider the model proposed in Land et al. (2012a), where the active tension *T*_*a*_ depends on the intracellular calcium concentration *Ca*_*i*_, the fiber stretch $\lambda =\sqrt{{\hat{\text{a}}}_{l}^{T}\text{C}{\hat{\text{a}}}_{l}}$, the fiber stretch-rate $\frac{d\lambda }{dt}$ and auxiliary variables included in vector **z**, i.e.,

$$\begin{array}{l} \{\begin{array}{l} \frac{d\text{z}}{dt}=R_{z}\left(\text{z},Ca_{i},\lambda ,\frac{d\lambda }{dt}\;\right)\\ T_{a}=f_{Ta}\left(\text{z},\lambda ,\frac{d\lambda }{dt}\right). \end{array} \end{array}$$

The generated active force is assumed to act only along the fiber direction, so the active Cauchy stress is

$$\begin{array}{l} \sigma^{act}\left(\text{x},t\right)=T_{a}\;{\text{a}}_{l}\left(\text{x}\right)⊗{\text{a}}_{l}\left(\text{x}\right), \end{array}$$

where **a**_*l*_ is a unit vector parallel to the local fiber direction and *T*_*a*_ is the active fiber stress associated to the deformed cardiac tissue. In the deformed configuration, the unit vector parallel to the local fiber direction can be written as

(4) $$\begin{array}{l} {\text{a}}_{l}=\frac{\text{F}{\hat{\text{a}}}_{l}}{\left||\text{F}{\hat{\text{a}}}_{l}|\right|}=\frac{\text{F}{\hat{\text{a}}}_{l}}{\sqrt{{\hat{\text{a}}}_{l}^{T}\text{C}{\hat{\text{a}}}_{l}}}, \end{array}$$

where ${\hat{\text{a}}}_{l}$ is the fiber direction in the reference configuration. Then the active stress component **S**^*act*^ of the second Piola-Kirchhoff tensor is given by

$$\begin{array}{l} {\text{S}}^{act}=J\;{\text{F}}^{-1}\sigma^{act}{\text{F}}^{-T}=J\;T_{a}\frac{{\hat{\text{a}}}_{l}⊗{\hat{\text{a}}}_{l}}{{\hat{\text{a}}}_{l}^{T}\text{C}{\hat{\text{a}}}_{l}}. \end{array}$$

### 2.3. The bioelectrical bidomain model

We denote by *v*, *u*_*e*_, **w**, **c** the transmembrane potential, the extracellular potential, the gating and ionic concentrations variables on the deformed configuration and by $\hat{v}$, ${\hat{u}}_{e}$, $\hat{\text{w}}$, $\hat{\text{c}}$ the same quantities on reference configuration. The Bidomain model, written on the deformed configuration Ω(*t*) is given in its parabolic-elliptic formulation by

(5) $$\begin{array}{l} \{\begin{array}{l} c_{m}\frac{\partial v}{\partial t}-\text{div}(D_{i}\nabla (v+u_{e}))+i_{ion}(v,\text{w},\text{c},\lambda )=i_{app}^{i}\\ -\text{div}(D_{i}\nabla v)-\text{div}((D_{i}+D_{e})\nabla u_{e})=i_{app}^{i}+i_{app}^{e}, \end{array} \end{array}$$

where *c*_*m*_ and *i*_*ion*_ are the membrane capacitance and ionic current per unit volume, respectively. We apply insulating boundary conditions on ∂Ω(*t*), i.e.,

$${\text{n}}^{T}D_{i}\nabla \left(v+u_{e}\right)=0\text{ and }{\text{n}}^{T}D_{e}\nabla u_{e}=0,$$

with **n** being the normal to ∂Ω(*t*). In order to satisfty the compatibility condition $\int_{\Omega \left(t\right)}\left(i_{app}^{i}+i_{app}^{e}\right)d\text{x}=0$, we choose $i_{app}^{i}=-i_{app}^{e}=i_{app}$; see e.g., Colli Franzone et al. (2014). In the Lagrangian framework, after the pull-back on the reference configuration $\hat{\Omega }\times \left(0,T\right)$, this system becomes

(6) $$\begin{array}{l} \{\begin{array}{l} c_{m}J\left(\frac{\partial \hat{v}}{\partial t}-F^{-T}\text{Grad}\hat{v}\;\cdot \;V\right)-\text{Div}(JF^{-1}{\hat{D}}_{i}F^{-T}\text{Grad}(\hat{v}+{\hat{u}}_{e}))\\ +\;Ji_{ion}(\hat{v},\hat{\text{w}},\hat{\text{c}},\lambda )=J{\hat{i}}_{app},\\ -\text{Div}(J\;F^{-1}{\hat{D}}_{i}F^{-T}\text{Grad}\hat{v})-\text{Div}(J\;F^{-1}({\hat{D}}_{i}+{\hat{D}}_{e})F^{-T}\text{Grad}{\hat{u}}_{e})=0, \end{array} \end{array}$$

where $\text{V}=\frac{\partial \text{u}}{\partial t}$ is the rate of deformation; see Colli Franzone et al. (2016a) for the detailed derivation. These two partial differential equations (PDEs) are coupled through the reaction term *i*_*ion*_ with the ODE system of the membrane model, given in Ω(*t*) × (0, *T*) by

(7) $$\begin{array}{l} \frac{\partial \text{w}}{\partial t}-{\text{R}}_{w}\left(v,\text{w}\right)=0,\;\frac{\partial \text{c}}{\partial t}-{\text{R}}_{c}\left(v,\text{w},\text{c}\right)=0. \end{array}$$

The bioelectrical system (Equations 6, 7) is completed by prescribing initial conditions on $\hat{v},\text{w},\text{c}$, insulating boundary conditions on ${\hat{u}}_{e},{\hat{u}}_{i}=\hat{v}+{\hat{u}}_{e}$, and the intra- and extracellular applied current ${\hat{i}}_{app}={\hat{i}}_{app}^{i}=-{\hat{i}}_{app}^{e}$. We recall that the extracellular potential ${\hat{u}}_{e}$ is defined only up to a time dependent constant in space *R*(*t*), which can be determined by choosing a reference potential. Here we select as a reference potential the average of the extracellular potential over the cardiac volume, i.e., we require $\int_{\hat{\Omega }}{\hat{u}}_{e}\left(\text{X},t\right)J\left(\text{X},t\right)d\text{X}=0$. Assuming transversely isotropic properties of the intra- and extracellular media, the conductivity tensors on the deformed configuration are given by

$$\begin{array}{l} D_{i,e}=\sigma_{t}^{i,e}I+\left(\sigma_{l}^{i,e}-\sigma_{t}^{i,e}\right){\text{a}}_{l}⊗{\text{a}}_{l}, \end{array}$$

where $\sigma_{l}^{i,e},\;\sigma_{t}^{i,e}$ are the the intra- and extracellular conductivity coefficients measured along the fiber direction **a**_*l*_ and any cross fiber direction, respectively. From Equation (4), it follows that the tensors *D*_*i,e*_(*x, t*) written on the reference configuration are

(8) $$\begin{array}{l} {\hat{D}}_{i,e}\left(\text{X},t\right)=D_{i,e}\left(\text{x}\left(\text{X},t\right),t\right)=\sigma_{t}^{i,e}I+\left(\sigma_{l}^{i,e}-\sigma_{t}^{i,e}\right)\frac{\text{F}{\hat{\text{a}}}_{l}{\hat{\text{a}}}_{l}^{T}{\text{F}}^{T}}{{\hat{\text{a}}}_{l}^{T}\text{C}{\hat{\text{a}}}_{l}^{T}}. \end{array}$$

Therefore, the equivalent conductivity tensors appearing into the bidomain model written in the reference configuration are given by

(9) $$\begin{array}{l} \begin{array}{c} J{\text{F}}^{-1}{\hat{D}}_{i,e}\left(\text{X},t\right){\text{F}}^{-T}=\sigma_{t}^{i,e}\;{\text{C}}^{-1}+\left(\sigma_{l}^{i,e}-\sigma_{t}^{i,e}\right)\frac{{\hat{\text{a}}}_{l}{\hat{\text{a}}}_{l}^{T}}{{\hat{\text{a}}}_{l}^{T}\text{C}{\hat{\text{a}}}_{l}^{T}}. \end{array} \end{array}$$

For the values of the conductivity coefficients of the Bidoman model (see Colli Franzone et al., 2016a).

### 2.4. The ionic membrane model and stretch-activated channel currents

The ionic current in the Bidomain model (Equation 6) is given by *i*_*ion*_ = χ*I*_*ion*_, where χ is the membrane surface to volume ratio and the ionic current per unit area of the membrane surface *I*_*ion*_ is given by the sum $I_{ion}\left(v,\text{w},\text{c},\lambda \right)=I_{ion}^{m}\left(v,\text{w},\text{c}\right)+I_{sac}$ of two terms: the ionic term $I_{ion}^{m}\left(v,\text{w},\text{c}\right)$ given by the ten Tusscher model (TP06) (ten Tusscher et al., 2004; ten Tusscher and Panfilov, 2006), available from the cellML depository (models.cellml.org/cellml), and a stretch-activated current term *I*_*sac*_. The TP06 ionic model also specifies the functions *R*_*w*_(*v*, **w**) and *R*_*c*_(*v*, **w**, **c**) in the ODE system Equation (Equation 7), consisting of 17 ordinary differential equations modeling the main ionic currents dynamics.

The stretch-activated current (SAC) is modeled as the sum of a non-selective and a potassium selective currents

$$I_{sac}=I_{ns}+I_{Ko},$$

as in Niederer and Smith (2007). The non-selective SAC current is defined by

$$I_{ns}=I_{ns,Na}+I_{ns,K}=g_{ns}\;\gamma_{sl}\left(\lambda \right)\;\left[r\;\left(v-v_{Na}\right)\;+\left(v-v_{K}\right)\right],$$

with γ_*sl*_(λ) = 10*max*(λ−1, 0), $g_{ns}=4.13\cdot 10^{-3}\;mS/{\text{cm}}^{2}$ and the value of *r* measures the relative conductance of the ions *Na*^+^ and *K*^+^ and determines the reversal potential *v*_*ns*_ of *I*_*ns*_, varying the degree of expression of the ions *Na*^+^ and *K*^+^. We have chosen *r* = 0.2.

The *K*^+^ selective SAC current is defined by

$$I_{Ko}=g_{Ko}\frac{\gamma_{SL,Ko}}{1+exp\left(-\left(10+v\right)/45\right)}\left(v-v_{K}\right),$$

where $g_{Ko}=1.2\cdot 10^{-2}\;mS/{\text{cm}}^{2}$ and γ_*SL,Ko*_ = 3max(λ − 1, 0) + 0.7.

## 3. Numerical methods

### 3.1. Space and time discretization

#### 3.1.1. Domain geometry

We consider an idealized left ventricular geometry $\hat{\Omega }=\Omega \left(0\right)$ modeled as a truncated ellipsoid described in ellipsoidal coordinates by the parametric equations

$$\{\begin{array}{ll} x=a(r)\cos\theta \cos\phi \; & \phi_{min}\leq \phi \leq \phi_{max},\\ y=b(r)\cos\theta \sin\phi \; & \theta_{min}\leq \theta \leq \theta_{max},\\ z=c(r)\sin\theta \; & 0\leq r\leq 1. \end{array}$$

Here *a*(*r*) = *a*_1_ + *r*(*a*_2_ − *a*_1_), *b*(*r*) = *b*_1_ + *r*(*b*_2_ − *b*_1_), *c*(*r*) = *c*_1_ + *r*(*c*_2_ − *c*_1_), and *a*_1_ = *b*_1_ = 1.5, *a*_2_ = *b*_2_ = 2.7, *c*_1_ = 4.4, *c*_2_ = 5 (all in *cm*) and ϕ_*min*_ = − π/2, ϕ_*max*_ = 3π/2, θ_*min*_ = − 3π/8, θ_*max*_ = π/8. We will refer to the inner surface of the truncated ellipsoid (*r* = 0) as endocardium and to the outer surface (*r* = 1) as epicardium. Proceeding counterclockwise from epicardium to endocardium, the cardiac fibers rotate intramurally linearly with the depth, for a total amount of 120°. Considering a local ellipsoidal reference system (**e**_ϕ_, **e**_θ_, **e**_*r*_), the fiber direction **a**_*l*_(**x**) at a point **x** is given by **a**_*l*_(**x**) = **b**_*l*_(**x**)cos(β) + **n**(**x**)cos(β), where

$$\begin{array}{l} {\text{b}}_{l}\left(\text{x}\right)={\text{e}}_{\phi }\cos\alpha \left(r\right)+{\text{e}}_{\theta }\sin\alpha \left(r\right),\text{ with}\\ \alpha \left(r\right)=\frac{2}{3}\pi \left(1-r\right)-\frac{\pi }{4},\;0\leq r\leq 1, \end{array}$$

**n**(**x**) is the unit outward normal to the ellipsoidal surface at **x** and β is the imbrication angle given by β = arctan(cosα tanγ), with γ = θ(1 − *r*)60/π.

#### 3.1.2. Time discretization

The time discretization of the electromechanical model is performed by the following semi-implicit splitting method, where different electrical and mechanical time steps could be used.

(a) given *v*^*n*^, **w**^*n*^, **c**^*n*^ at time step *t*_*n*_, we compute the new variables **w**^*n*+1^, **c**^*n*+1^ by solving the ODE system of the ionic membrane model (Equation 7) with a first order implicit-explicit (IMEX) method, i.e.,

$$\{\begin{array}{l} \frac{{\text{w}}^{n+1}-{\text{w}}^{n}}{\Delta t}-{\text{R}}_{w}(v^{n},{\text{w}}^{n+1})=0,\\ \frac{{\text{c}}^{n+1}-{\text{c}}^{n}}{\Delta t}-{\text{R}}_{c}(v^{n},{\text{w}}^{n+1},{\text{c}}^{n})=0; \end{array}$$

(b) given the calcium concentration $Ca_{i}^{n+1}$, which is part of the vector of concentration variables **c**^*n*+1^, we compute the new deformed coordinates **x**^*n*+1^, providing the new deformation gradient tensor **F**_*n*+1_, by solving the variational formulation of the mechanical problem (Equation 1) and the active tension system, i.e.,

$$\{\begin{array}{l} {\text{z}}^{n+1}={\text{z}}^{n}+\Delta tR_{z}\left({\text{z}}^{n+1},Ca_{i}^{n+1},\lambda^{n+1},\frac{\lambda^{n+1}-\lambda^{n}}{\Delta t_{n}}\;\right)\\ T_{a}^{n+1}=f_{Ta}\left({\text{z}}^{n+1},\lambda^{n+1},\frac{\lambda^{n+1}-\lambda^{n}}{\Delta t_{n}}\right)\\ \text{Div}({\text{F}}_{n+1}{\text{S}}_{n+1})=0; \end{array}$$

(c) given **w**^*n*+1^, **c**^*n*+1^, **F**_*n*+1_ and *J*_*n*+1_ = det(**F**_*n*+1_), we compute the new electric potentials $v^{n+1},\;u_{e}^{n+1}$ by solving the variational formulation of the Bidomain system (Equation 6) with a first order IMEX and operator splitting method, consisting of decoupling the parabolic from the elliptic equation, i.e.,

$$\begin{array}{l} \{\begin{array}{l} -\text{Div}(J^{n+1}\;F_{n+1}^{-1}{\hat{D}}_{i}F_{n+1}^{-T}\text{ Grad}{\hat{v}}^{n})-\text{Div}(J_{n+1}\;F_{n+1}^{-1}({\hat{D}}_{i}+{\hat{D}}_{e})F_{n+1}^{-T}\text{ Grad}{\hat{u}}_{e}^{n})=0,\\ c_{m}J_{n+1}\left(\frac{{\hat{v}}^{n+1}-{\hat{v}}^{n}}{\Delta t}-F_{n+1}^{-T}\text{Grad}{\hat{v}}^{n}\cdot V^{n+1}\right)-\text{Div}(J_{n+1}F_{n+1}^{-1}{\hat{D}}_{i}F_{n+1}^{-T}\text{ Grad}({\hat{v}}^{n+1}+{\hat{u}}_{e}^{n+1}))+\\ J_{n+1}i_{ion}({\hat{v}}^{n},{\hat{\text{w}}}^{n+1},{\hat{\text{c}}}^{n+1},\lambda^{n+1})=J_{n+1}\;{\hat{i}}_{app}^{n+1}. \end{array} \end{array}$$

In our simulations, we use the electrical time step size Δ_*e*_*t* = 0.05 *ms*, and a mechanical times step five times larger, Δ_*m*_*t* = 0.25 *ms*. In order to approximate the convective term in the variational formulation of Equation (6), an *upwind* discretization strategy is employed. We refer to Colli Franzone et al. (2015) and Colli Franzone et al. (2016a) for more details about the numerical scheme.

#### 3.1.3. Space discretization

The cardiac domain is discretized with a structured hexahedral grid *T*_*h*_*m*__ for the mechanical model (Equation 1) and *T*_*h*_*e*__ for the Bidomain model (Equation 6), where *T*_*h*_*e*__ is a refinement of *T*_*h*_*m*__, i.e., the mechanical mesh size *h*_*m*_ is an integer multiple of the electrical mesh size *h*_*e*_. We consider the variational formulations of both mechanical and bioelectrical models and then approximate all scalar and vector fields by isoparametric *Q*_1_ finite elements in space. In all our simulations, we employ an electrical mesh size *h*_*e*_ = 0.01 cm in order to properly resolve the sharp excitation front, while the smoother mechanical deformation allow us to use a coarse mechanical mesh of size *h*_*m*_ = 0.08 cm. The resulting electrical mesh consists of *N*_ϕ_ × *N*_θ_ × *N*_*k*_ elements, whose values will be specified in each numerical test reported in the Results section.

### 3.2. Computational kernels and parallel solvers

At each time step of the space—time discretization described above, the two main computational kernels are:

(a) the solution of a non-linear system arising from the discretization of the mechanical problem (1); to this end, we use a parallel Newton-Krylov-BDDC (NK-BDDC) solver, where the Krylov method chosen is GMRES and the BDDC preconditioner will be described in the next sections;

(b) the solution of two linear systems deriving from the discretization of the elliptic and parabolic equations in the Bidomain model (Equation 6); to this eand, we use a parallel Preconditioned Conjugate Gradient (PCG) method, with Multilevel Additive Schwarz preconditioner for the very ill-conditioned elliptic system and with Block-Jacobi preconditioner for the easier parabolic system.

The parallelization of these two main computational kernels of our electro-mechanical solver is based on the parallel library PETSc (Balay et al., 2012) from the Argonne National Laboratory. All the parallel simulations have been performed on high-performance supercomputers and Linux clusters described in the Result section. For the parallel implementation of the BDDC preconditioner, see Zampini (2016).

### 3.3. Multilevel Additive Schwarz preconditioners

We now describe the Multilevel Additive Schwarz preconditioner employed in the PCG solution of the elliptic kernel (b) associated with the Bidomain system. Let Ω^*k*^, *k* = 0, …, ℓ − 1 be a family of ℓ nested triangulations of Ω, with finer mesh sizes from level 0 to ℓ − 1, and let A^*k*^ be the matrix obtained by discretizing the second equation of Equation (6) on Ω^*k*^; we have ${\text{A}}^{\ell -1}=A_{bid}$, where *A*_*bid*_ is the stiffness matrix related to the elliptic equation of Equation (6) discretized on the fne mesh. Denote by R^*k*^ the restriction operators from Ω^ℓ − 1^ to Ω^*k*^. We decompose each grid Ω^*k*^, for *k* = 1, …, ℓ − 1, into *N*_*k*_ overlapping subgrids $\Omega_{i}^{k}$ for *i* = 1, …, *N*_*k*_, such that the overlap size δ^*k*^ at level *k* = 1, …, ℓ − 1 equals the mesh size *h*^*k*^ of the grid Ω^*k*^. We denote by $R_{i}^{k}$ the restriction operator from Ω^ℓ − 1^ to $\Omega_{i}^{k}$ and define ${\text{A}}_{i}^{k}:={\text{R}}_{i}^{k}{\text{A}}^{k}{\text{R}}_{i}^{k^{T}}$. The Multilevel Additive Schwarz (MAS(ℓ)) preconditioner is given by

$$B_{MAS}^{-1}:={\text{R}}^{0^{T}}{\text{A}}^{0^{-1}}{\text{R}}^{0}+\overset{\ell -1}{\underset{k=1}{\sum }}\overset{N_{k}}{\underset{i=1}{\sum }}{\text{R}}_{i}^{k^{T}}{\text{A}}_{i}^{k^{-1}}{\text{R}}_{i}^{k}.$$

The resulting PCG algorithm has a convergence rate independent of the number of subdomains *N*_*k*_ (scalability), the number of levels ℓ (multilevel optimality), while it depends linearly on the ratio *H*_*k*_/*h*_*k*_ of subdomain to element size on level *k* (optimality); see Pavarino and Scacchi (2008), Scacchi (2008), and Pavarino and Scacchi (2011) for the theoretical details.

### 3.4. Iterative substructuring, Schur complement system and BDDC preconditioners

We then turn to the BDDC preconditioner used in the mechanical computational kernel (a) above, i.e., the Jacobian system arising at each iteration of the Newton method applied to the non-linear elasticity system (Equation 1). For sake of simplicity, in the following sections we will denote the reference domain by Ω instead of $\hat{\Omega }$. We consider a decomposition of Ω into *N* non-overlapping subdomains Ω_*i*_ of diameter *H*_*i*_

$$\Omega =\overset{N}{\underset{i=1}{⋃}}\Omega_{i},$$

and set *H* = max *H*_*i*_. We first reduce the Jacobian system

(10) $$\begin{array}{l} Kx=f, \end{array}$$

arising at each Newton step of the mechanical solver, to the interface

$$\Gamma :=(\overset{N}{\underset{i=1}{⋃}}\partial \Omega_{i})\backslash \partial \Omega ,$$

by eliminating the interior degrees of freedom (dofs) associated with the basis functions having support in each subdomain's interior and obtaining the Schur complement system

(11) $$\begin{array}{l} S_{\Gamma }x_{\Gamma }=g_{\Gamma }. \end{array}$$

Here $S_{\Gamma }=K_{\Gamma \Gamma }-K_{\Gamma I}K_{II}^{-1}K_{\Gamma I}$ and $\;g_{\Gamma }=f_{\Gamma }-K_{\Gamma I}K_{II}^{-1}f_{I}$ are obtained from the global system (Equation 10) by reordering the finite element basis functions into interior (denoted by the subscript *I*) and interface (denoted by the subscript Γ) basis functions

(12) $$\begin{array}{l} \left(\begin{array}{cc} K_{II} & K_{I\Gamma }\\ K_{\Gamma I} & K_{\Gamma \Gamma } \end{array}\right)\left(\begin{array}{c} x_{I}\\ x_{\Gamma } \end{array}\right)=\left(\begin{array}{c} f_{I}\\ f_{\Gamma } \end{array}\right). \end{array}$$

The Schur complement system (Equation 11) is solved iteratively by the GMRES method, where only the action of *S*_Γ_ on a given vector is required and *S*_Γ_ is never explicitly formed; instead, a block diagonal problem on the interior dofs is solved while computing the matrix vector product. Once the interface solution *x*_Γ_ has been determined, the internior dofs *x*_*I*_ can be found by solving local problems on each subdomain Ω_*i*_. We then solve by the GMRES method the preconditioned Schur complement system

(13) $$\begin{array}{l} M_{\text{BDDC}}^{-1}S_{\Gamma }x_{\Gamma }=M_{\text{BDDC}}^{-1}g_{\Gamma }, \end{array}$$

where $M_{\text{BDDC}}^{-1}$ is the BDDC preconditioner, defined in Equation (17) below.

Balanced Domain Decomposition by Constraints (BDDC) preconditioners where introduced by Dohrmann (2003) and first analyzed by Mandel and Dohrmann (2003) and Mandel et al. (2005). In these methods all local and coarse problems are treated additively and the user selects the so-called primal continuity constraints across the subdomains' interface. Usual choices of primal constraints are e.g., point constraints at subdomain vertices and/or averages or moments over subdomains edges or faces. Closely related to BDDC methods are FETI and FETI-DP algorithms, as well as the previous balancing Neumann-Neumann methods; for more details, we refer the ineterested reader to the domain decomposition monograph (Toselli and Widlund, 2005, Ch. 6). See also Brands et al. (2008) and Klawonn and Rheinbach (2010) for FETI-DP algorithms applied in other fields of computational biomechanics.

#### 3.4.1. Subspace decompositions

Let *V* be the *Q*_1_ finite element space for displacements and *V*^(*i*)^ be the local finite element space defined on subdomain Ω_*i*_ that vanish on ∂Ω_*i*_ ∩ ∂Ω_*D*_. This local space can be split into a direct sum of its interior (I) and interface (Γ) subspaces $V^{\left(i\right)}=V_{I}^{\left(i\right)}⊕V_{\Gamma }^{\left(i\right)}$ and we can define the associated product spaces as

$$V_{I}:=\overset{N}{\underset{i=1}{\prod }}V_{I}^{\left(i\right)},\;V_{\Gamma }:=\overset{N}{\underset{i=1}{\prod }}V_{\Gamma }^{\left(i\right)}.$$

While our finite element approximations are continuous across the interface Γ, the functions of *V*_Γ_ are generally discontinuous across Γ, We then define the subspace

$${\hat{V}}_{\Gamma }:=\left\{\text{functions of }V_{\Gamma }\text{ that are continuous across}\Gamma \right\},$$

and the intermediate subspace

$${\tilde{V}}_{\Gamma }:=V_{\Delta }⊕{\hat{V}}_{\Pi },$$

defined by further splitting the interface dofs (denoted by the subscript Γ) into primal (subscript Π) and dual (subscript Δ) dofs. Here:

(a) the subspace ${\hat{V}}_{\Pi }$ consists of functions which are continuous at selected *primal* variables. These can be e.g., the subdomain basis functions associated with subdomains' vertices and/or edge/face basis functions with constant values at the nodes of the associated edge/face. A change of basis can be performed so that each primal variable correspond to an explicit dof.

(b) the subspace $V_{\Delta }=\prod_{i=1}^{N}V_{\Delta }^{\left(i\right)}$ is the product space of the local subspaces $V_{\Delta }^{\left(i\right)}$ of *dual* interface functions that vanish at the primal dofs.

#### 3.4.2. Restriction and scaling operators

The definition of our dual-primal preconditioners require also the following restriction and interpolation operators, associated with boolean matrices (with {0, 1} elements):

(14) $$\begin{array}{l} \begin{array}{cc} R_{\Gamma \Delta }:{\tilde{V}}_{\Gamma }\to V_{\Delta }, & \;R_{\Gamma \Pi }:{\tilde{V}}_{\Gamma }\to {\hat{V}}_{\Pi },\\ R_{\Delta }^{\left(i\right)}:V_{\Delta }\to V_{\Delta }^{\left(i\right)}, & \;R_{\Pi }^{\left(i\right)}:{\hat{V}}_{\Pi }\to {\hat{V}}_{\Pi }^{\left(i\right)}, \end{array} \end{array}$$

where ${\hat{V}}_{\Pi }^{\left(i\right)}$ is the local *primal* subspace. Moreover, we define the pseudo-inverse counting functions $\delta_{i}^{†}\left(x\right)$, which are defined at each dof *x* on the interface of subdomain Ω_*i*_ by

(15) $$\begin{array}{l} \delta_{i}^{†}\left(x\right):=\frac{1}{N_{x}}, \end{array}$$

with $N_{x}$ the number of subdomains sharing *x*. We finally define scaled local restriction operators $R_{D,\Delta }^{\left(i\right)}$ by scaling by by $\delta_{i}^{†}$ the only nonzero element of each row of $R_{\Delta }^{\left(i\right)}$. We then define the scaling matrix

(16) $$\begin{array}{l} R_{D,\Gamma }:=\text{the direct sum }R_{\Gamma \Pi }⊕R_{D,\Delta }^{\left(i\right)}R_{\Gamma \Delta }. \end{array}$$

#### 3.4.3. Choice of primal constraints

The efficiency of BDDC (and more in general dual-primal) preconditioners is strongly dependent of the choice of primal contraints. The simplest choice of selecting the subdomains vertices as primal dofs is not always sufficient to obtain scalable and fast preconditioners. Therefore, richer (and computationally more expensive) primal sets have been developed in order to obtain faster preconditioners. These stronger preconditioners are based on larger coarse problems employing also edge and/or face based primal dofs, see e.g., Toselli and Widlund (2005).

#### 3.4.4. Matrix form of the BDDC preconditioner

Analogously to the dual-primal splitting introduced before, we partition the local dofs into interior (I), dual (Δ), and primal (Π) dofs, so that the local stiffness matrix *K*^(*i*)^ associated to subdomain Ω_*i*_ can be written as

$$K^{\left(i\right)}=\left[\begin{array}{cc} K_{II}^{\left(i\right)} & K_{\Gamma I}^{{\left(i\right)}^{T}}\\ K_{\Gamma I}^{\left(i\right)} & K_{\Gamma \Gamma }^{\left(i\right)} \end{array}\right]=\left[\begin{array}{ccc} K_{II}^{\left(i\right)} & K_{\Delta I}^{{\left(i\right)}^{T}} & K_{\Pi I}^{{\left(i\right)}^{T}}\\ K_{\Delta I}^{\left(i\right)} & K_{\Delta \Delta }^{\left(i\right)} & K_{\Pi \Delta }^{{\left(i\right)}^{T}}\\ K_{\Pi I}^{\left(i\right)} & K_{\Pi \Delta }^{\left(i\right)} & K_{\Pi \Pi } \end{array}\right].$$

The BDDC preconditioner is then defined as

(17) $$\begin{array}{l} M_{\text{BDDC}}^{-1}=R_{D,\Gamma }^{T}{\tilde{S}}_{\Gamma }^{-1}R_{D,\Gamma }, \end{array}$$

where the scaled restriction matrix *R*_*D*,Γ_ has been defined in Equations (14, 16), and

(18) $$\begin{array}{l} {\tilde{S}}_{\Gamma }^{-1}=R_{\Gamma \Delta }^{T}\left(\overset{N}{\underset{i=1}{\sum }}\left[\begin{array}{cc} 0 & R_{\Delta }^{{\left(i\right)}^{T}} \end{array}\right]{\left[\begin{array}{cc} K_{II}^{\left(i\right)} & K_{\Delta I}^{{\left(i\right)}^{T}}\\ K_{\Delta I}^{\left(i\right)} & K_{\Delta \Delta }^{\left(i\right)} \end{array}\right]}^{-1}\left[\begin{array}{c} 0\\ R_{\Delta }^{\left(i\right)} \end{array}\right]\right)R_{\Gamma \Delta }+\Phi S_{\Pi \Pi }^{-1}\Phi^{T}. \end{array}$$

The first term in Equation (18) represent the sum of local problems on each subdomain Ω_*i*_, with Neumann data on the local dual dofs and with zero Dirichlet data on the local primal dofs. The second term in Equation (18) represents a coarse problem for the primal variables involving the coarse matrix

$$\begin{array}{l} S_{\Pi \Pi }=\overset{N}{\underset{i=1}{\sum }}R_{\Pi }^{{\left(i\right)}^{T}}\left(K_{\Pi \Pi }^{\left(i\right)}-\left[\begin{array}{cc} K_{\Pi I}^{\left(i\right)} & K_{\Pi \Delta }^{\left(i\right)} \end{array}\right]{\left[\begin{array}{cc} K_{II}^{\left(i\right)} & K_{\Delta I}^{{\left(i\right)}^{T}}\\ K_{\Delta I}^{\left(i\right)} & K_{\Delta \Delta }^{\left(i\right)} \end{array}\right]}^{-1}\left[\begin{array}{c} K_{\Pi I}^{{\left(i\right)}^{T}}\\ K_{\Pi \Delta }^{{\left(i\right)}^{T}} \end{array}\right]\right)R_{\Pi }^{\left(i\right)} \end{array}$$

and a matrix Φ mapping primal to interface dofs

$$\Phi =R_{\Gamma \Pi }^{T}-R_{\Gamma \Delta }^{T}\overset{N}{\underset{i=1}{\sum }}\left[\begin{array}{cc} 0 & R_{\Delta }^{{\left(i\right)}^{T}} \end{array}\right]{\left[\begin{array}{cc} K_{II}^{\left(i\right)} & K_{\Delta I}^{{\left(i\right)}^{T}}\\ K_{\Delta I}^{\left(i\right)} & K_{\Delta \Delta }^{\left(i\right)} \end{array}\right]}^{-1}\left[\begin{array}{c} K_{\Pi I}^{{\left(i\right)}^{T}}\\ K_{\Pi \Delta }^{{\left(i\right)}^{T}} \end{array}\right]R_{\Pi }^{\left(i\right)}.$$

The columns of Φ are associated with coarse basis functions defined as the minimum energy extension into the subdomains with respect to the original bilinear form and subject to the chosen set of primal constraints.

For compressible linear elasticity problems it can be shown that the BDDC algorithm is scalable and quasi-optimal, satisfying a condition number bound (see e.g., Toselli and Widlund, 2005, Ch. 6.4) as

$$cond\left(M_{\text{BDDC}}^{-1}S_{\Gamma }\right)\leq C(\frac{H}{h})(1+\log\frac{H}{h}{)}^{2},$$

with $C\left(\frac{H}{h_{m}}\right)=\alpha$ constant if the primal space is sufficiently rich, while $C\left(\frac{H}{h}\right)=\alpha \frac{H}{h}$ if the primal space is the minimal one spanned by the dofs associated with the subdomain vertices. We recall that *H* is the characteristic subdomain size and *h* = *h*_*m*_ is the characteristic mechanical mesh size defined in section 3.1. We could not prove a similar bound for the convergence rate of our non-symmetric NK-BDDC preconditioned operator, since our complex non-linear elasticity problem (Equation 1) involves an exponential strain energy function. Nevertheless, the numerical results presented in the next section suggests that such a bound holds also for our operator and demonstrate the effectiveness and scalability of the NK-BDDC method.

## 4. Results

In this section, we report the results of several 3D parallel simulations with our electro-mechanical Bidomain solver, using two HPC architectures:

- the Marconi-A2 supercomputer of the Cineca Lab (http://www.hpc.cineca.it/hardware/marconi), an Intel OmniPath cluster with 3,600 nodes, each with 68 1.40 GHz Intel Xeon Phi 7250 Knights Landing (KNL) cores and 16 GB/node, for a total 244.800 cores;
- the Mira BG/Q supercomputer of the Argonne National Lab (https://www.alcf.anl.gov/mira), an IBM BG/Q machine with 49,152 nodes, each with 16 1.60 GHz PowerPC A2 cores and 16 GB/node, for a total 786,432 cores.

### 4.1. Test 1: double reentry simulation with the electro-mechanical bidomain model (Figures 2, 3)

**Figure 2:**
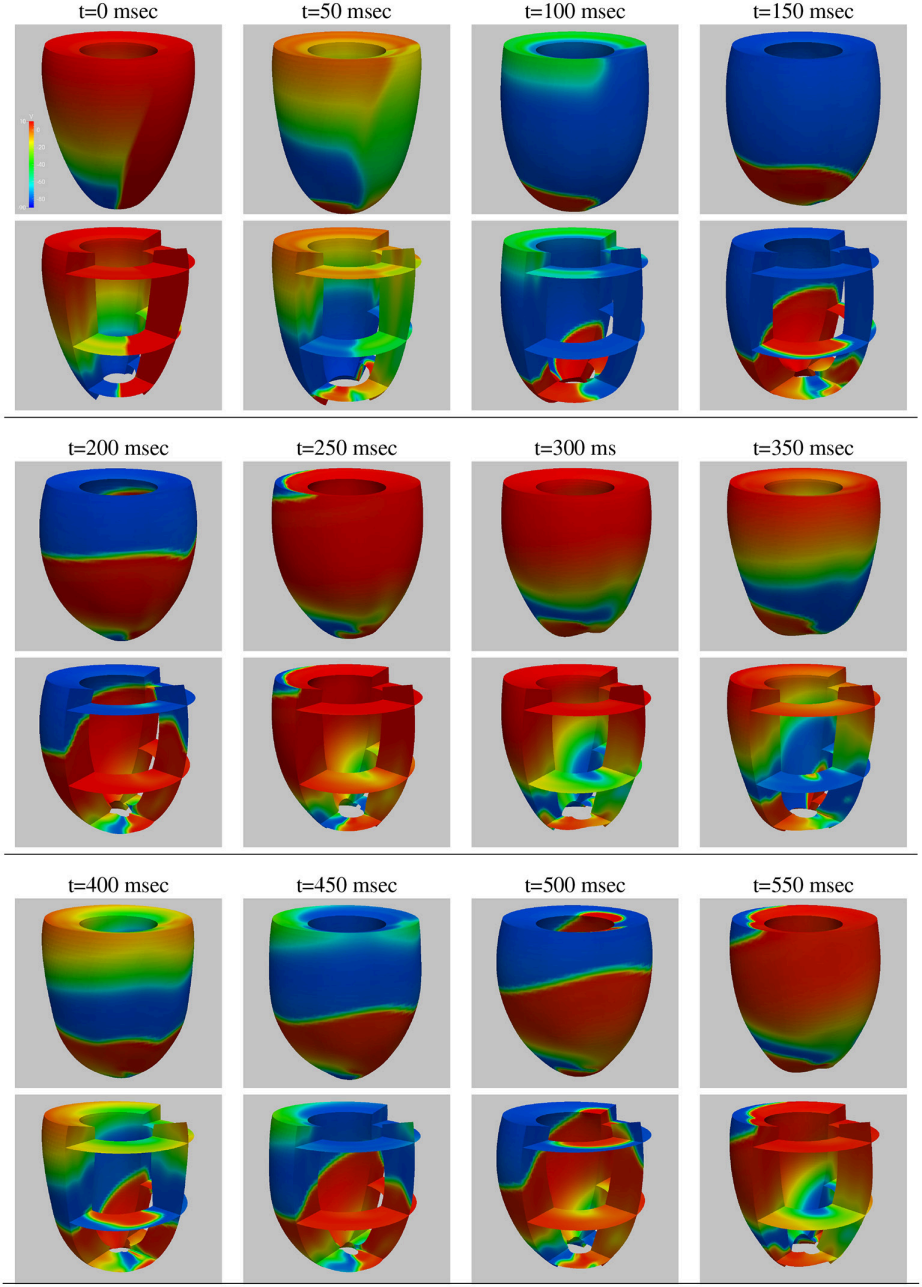
Test 1, double reentry simulation: snapshots (every 50 ms) of the transmembrane potential and mechanical deformation time evolution. At each time instant, we report the epicardial view **(Top)** and selected horizontal and vertical transmural sections **(Bottom)**.

We start by studying the performance of our electro-mechanical Bidomain solver on a closed ellipsoidal ventricular geometry during a double reentry dyamics initiated by an S1–S2 protocol. Figure 2 shows the snapshots of the transmembrane potential and mechanical deformation time evolution every 50 ms, computed on 256 KNL processors of Marconi-A2. At each time instant, we report the epicardial lateral view (top panel) and selected horizontal and vertical transmural sections (bottom panel). After three S1 stimulations applied at the apex every 500 ms (not shown), an S2 cross-gradient stimulation (visible as a vertical strip in the *t* = 0 panel) is applied 280 ms. after the last S1 stimulus, and this instant is taken as the reference time *t* = 0 ms for this simulation. Two counter-rotating scroll waves are generated by the S2 stimulus, with transmural filaments located near the apex and rotation period of about 250 ms (see the panels *t* = 0, 250, 500 ms). The lateral epicardial view of the upper panels shows mostly one of the two scroll waves, but the second almost-symmetric one is visible in the transmural sections of the lower panels.

**Figure 3:**
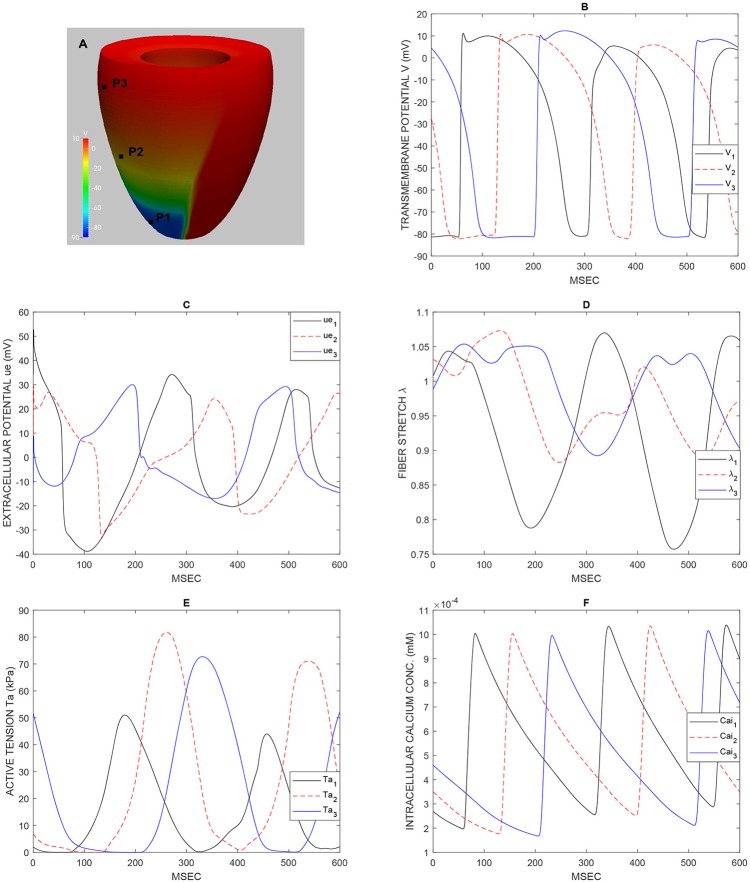
Test 1, double reentry simulation: waveforms at epicardial sites P1, P2, P3 shown in **(A)** of the transmembrane potential V **(B)**, extracellular potential *u*_*e*_ **(C)**, fiber stretch λ **(D)**, active tension *T*_*a*_ **(E)**, intracellular calcium concentration *Ca*_*i*_ **(F)**.

This reentry dynamics is visible also in Figure 3 that reports the waveforms at epicardial sites P1, P2, P3 (shown in Figure 3A) of the transmembrane potential V (Figure 3B), extracellular potential *u*_*e*_ (Figure 3C), fiber stretch λ (Figure 3D), active tension *T*_*a*_ (Figure 3E), intracellular calcium concentration *Ca*_*i*_ (Figure 3F).

### 4.2. Test 2: weak scalability of the elliptic bidomain - TP06 solver (Figures 4, 5)

**Figure 4:**
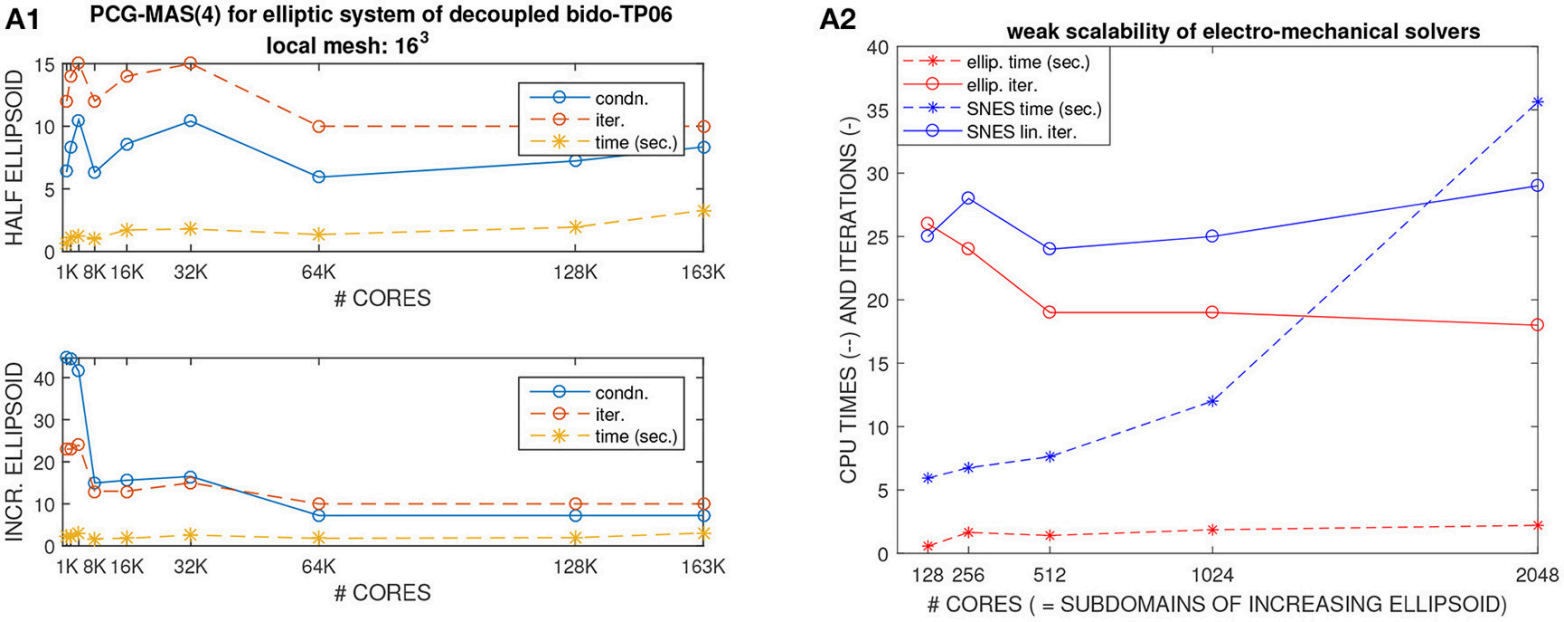
**(A1)** Test 2, weak scalability on MIRA BG/Q from 1K to 163K processors of the elliptic solver of the decoupled Bidomain - TP06 model. Condition number (blue), iteration counts (red), solution times (yellow) of PCG solver with Multilevel Additive Schwarz preconditioner. **(A2)** Test 3, weak scalability on Marconi-A2 from 128 to 2048 processors of the electro-mechanical solver (NK-BDDC). CPU times and iteration counts.

**Figure 5:**
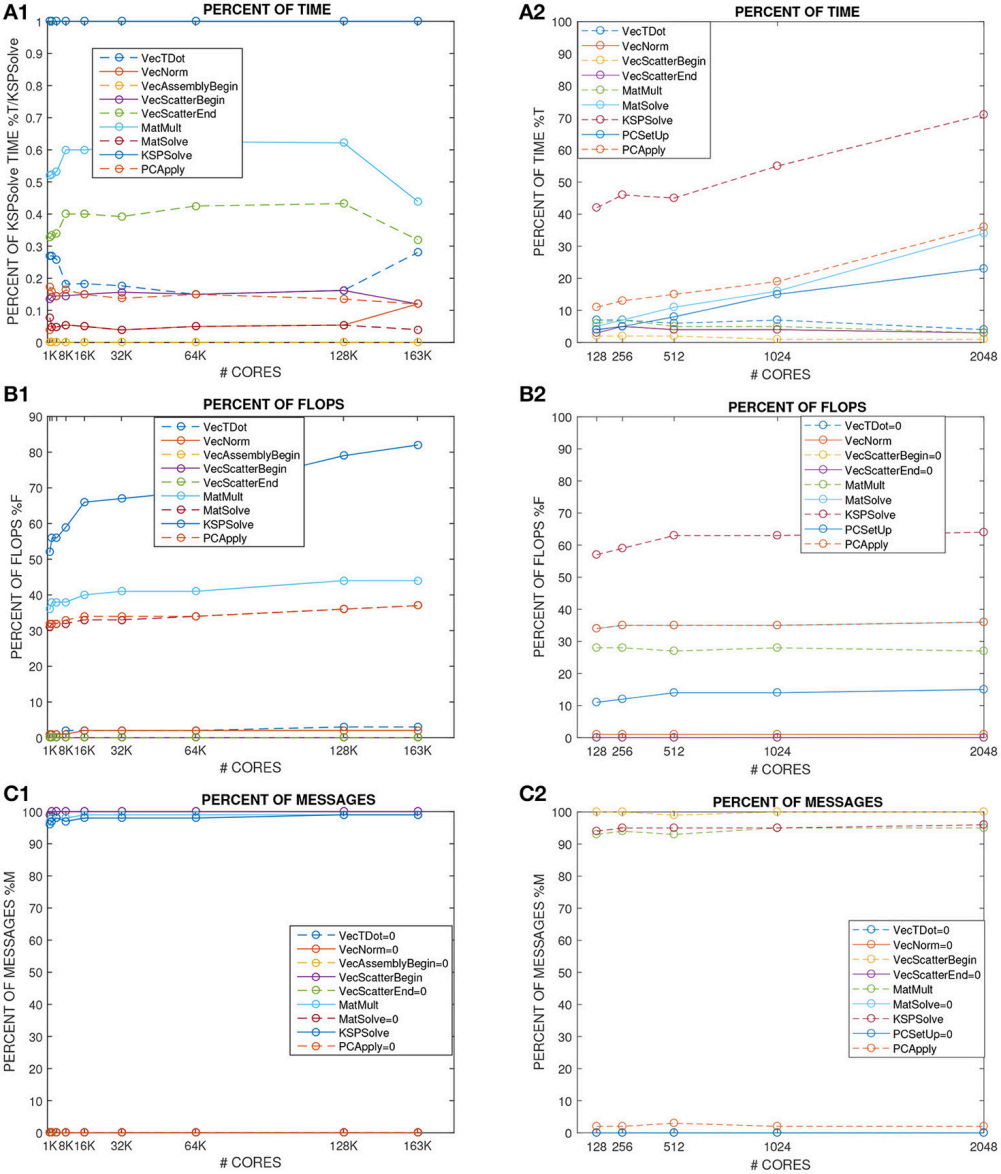
Left column Test 2, weak scalability on MIRA BG/Q from 1K to 163K processors of the elliptic solver of the decoupled Bidomain - TP06 model. Percent summary of time **(A1)**, flops **(B1)**, messages **(C1)** of the nine main PETSc functions (from VecTDot to PCApply) called by the elliptic solver. Right column Test 3, weak scalability on Marconi-A2 from 128 to 2048 processors of the electro-mechanical solver (NK-BDDC). Percent summary of time **(A2)**, flops **(B2)**, messages **(C2)** of the nine main PETSc functions (from VecTDot to PCApply) called by the elliptic solver.

Figures 4, 5 (left columns) report the results of weak scalability tests on MIRA BG/Q for the elliptic solver (PCG-MAS(4)) required by the bioelectrical Bidomain - TP06 model on a half ellipsoidal domain representing an idealized half left ventricle. The number of processors is increased from 1K to 163K cores of the Mira BG/Q supercomputer of the Argonne National Lab. Figure 4A1 reports the condition number (blue), iteration counts (red), solution times (yellow) of the PCG - MAS(4) solver. Both a fixed half ellipsoidal domain (Figure 4A1, top plot) and an increasing ellipsoidal domain (Figure 4A1, bottom plot) are considered, where in both cases the local meshsize (hence the local problem size on each processor) is kept fixed at *H*/*h* = 16. The results clearly show the very good scalability of the PCG - MAS(4) solver, since all quantities are bounded from above as the processor count is increased from 1K to 163K cores (a factor 163) and therefore the global problem size increases from about *O*(10^6^) to *O*(10^8^) degrees of freedom. In particular, we remark that in spite of this problem size increase of a factor 163, the CPU times are almost constant in the case of an increasing half ellipsoid (Figure 4A1, bottom plot) or increase by only a factor 2–3 in the case of a fixed half ellipsoid (Figure 4A1, top plot), while being almost constant between 16K and 128K cores.

Analogously, Figures 4, 5 (right columns) report the results of weak scalability tests on Marconi - A2 for the elliptic solver (PCG-MAS(4)) and also the non-linear mechanical solver (NK-BDDC), described in section 4.3 below. As before, the results clearly show the very good scalability of the PCG-MAS(4) solver, since all quantities associated with the elliptic solver are bounded from above.

In order to study more in detail the weak scalability test on a fixed half ellipsoid (Figure 4A1, top plot), we report in Figures 5A1,B1,C1 the percent summary (given by the LogView PETSc subroutine) of the main PETSc functions called by the PCG - MAS(4) elliptic solver. These PETSc functions, shown in the legend of each plot, range from inner products (VecTDoc) and vector norms (VecNorm) to the whole PCG solver (KSPSolve) and application of the MAS(4) preconditioner (PCApply). In particular, we report the percent of: CPU time as a fraction of the KSPSolve time (Figure 5A1), flops (Figure 5B1), messages (Figure 5C1). When one of these PETSc functions has a negligible percentage, the corresponding legend shows it equal to 0. After an initial increase in some cases, all reported quantities are very scalable up to 64K cores, and most up to 163K cores, except the VecTDot percent of flops (in Figure 5B1). As expected, the percentage of time (Figure 5A1) and flops (Figure 5B1) are dominated by the PCG solver (KSPSolve), followed by matrix multiplications (MatMult) and inner products (VecTDot). The percentage of messages (Figure 5C1) is dominated by vector scattering (VecScatterBegin), matrix multiplications (MatMult) and PCG (KSPSolve).

### 4.3. Test 3: weak scalability of the electro-mechanical solver (Figures 4, 5)

We then study the weak scalability of our electro-mechanical solver from 128 to 2048 KNL processors of Marconi-A2, in particular of the two main computational kernerls: the non-linear mechanical solver (NK-BDDC) and the linear elliptic Bidomain solver (PCG - MAS(4)). Figure 4A2 reports the CPU times and iteration counts for both solvers, while Figures 5A2,B2,C2 reports the percent summary of the main PETSc functions called by the electro-mechanical solver.

In this weak scaling test, the local meshsize (hence the local problem size on each processor) is kept fixed at *H*/*h* = 16, while the global problem size grows proportionally to the processor count by assigning one subdomain to each processor. Hence, the computational domain consists of increasing portions or an ellipsoidal domain. The results in Figure 4A2 clearly show the very good scalability of the PCG - MAS(4) elliptic linear solver, since both its CPU times and iteration counts are bounded from above as the processor count is increased to 2,048 cores. On the other hand, the timings of the non-linear SNES solver are not scalable beyond 512 processors, even if the iteration counts are. This is due to the non-scalability of the coarse solver (Mumps) employed in the BDDC preconditioner.

In order to study more in detail this scalability test, we report in Figures 5A2,B2,C2 the percent summary (given by the LogView PETSc subroutine) of the main PETSc functions called by the electro-mechanical solver. These PETSc functions, shown in the legend of each plot, range from inner products (VecTDoc) and vector norms (VecNorm) to the linear solvers (KSPSolve) and preconditioner applications (PCApply) required by both the linear (PCG-MAS(4)) and non-linear (NK-BDDC) solvers. In particular, we report the percent of: CPU time (Figures 5A2), flops (Figure 5B2) and messages (Figure 5C2). When one of these PETSc functions has a negligible percentage, the corresponding legend shows it equal to 0). All reported pertentages are very scalable, showing quite flat plots, except the time percentages (Figure 5A2), where the KSPSolve and PCApply percentages grow considerably beyond 512 cores, due mostly to the growth of MatSolve and PCSetUp, which we know already from Figures 5A1,A2 are due to the nonscalable direct coarse solve (Mumps) of the BDDC preconditioner called by the non-linear SNES solver. As expected, the percentage of time (Figure 5A2) and flops (Figure 5B2) are dominated by the PCG solver (KSPSolve), followed by PCApply and MatSolve. The percentage of messages (Figure 5C2) is dominated by vector scattering (VecScatterBegin), matrix multiplications (MatMult) and linear solves (KSPSolve).

### 4.4. Test 4: strong scalability of the non-linear electro-mechanical bidomain solver (Figures 6, 7)

**Figure 6:**
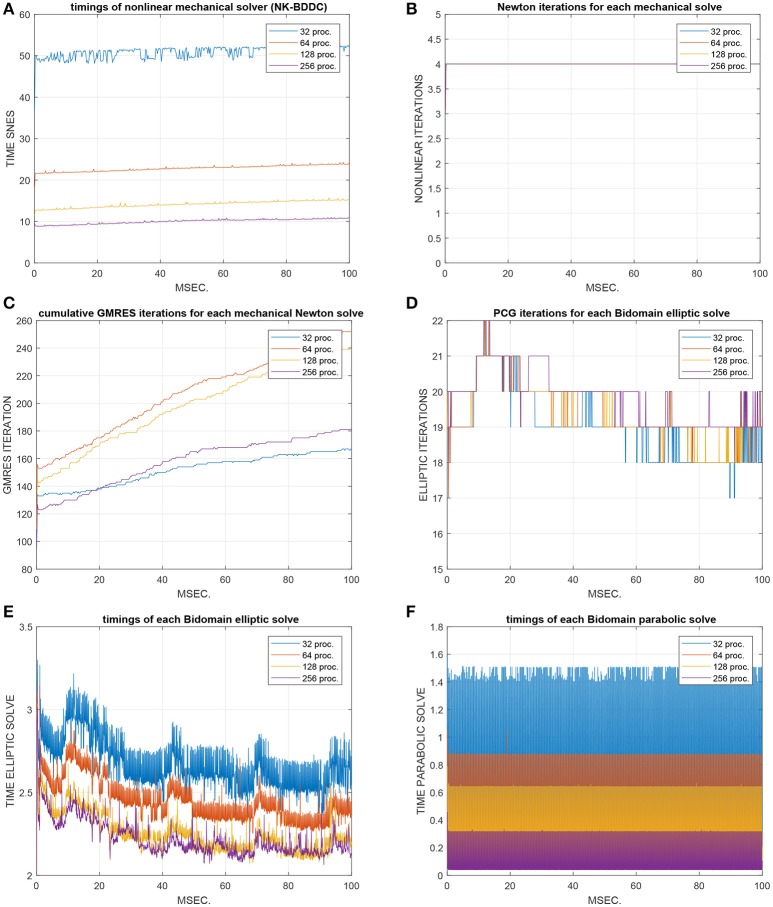
Test 4: time evolution over the [0 100] ms interval of CPU times and iterations of the nonlinear mechanical solver (NK-BDDC) in strong scalability tests from 32 to 256 processors of Marconi-A2. **(A)** timings of NK-BDDC solver. **(B)** Newton iterations for each NK-BDDC solve. **(C)** cumulative GMRES iterations for each NK-BDDC solve. **(D)** PCG iterations for each Bidomain elliptic solve. **(E)** timings of each Bidomain elliptic solve. **(F)** timings of each Bidomain parabolic solve.

Figure 6 reports the results of strong scalability tests on Marconi-A2 for the non-linear electro-mechanical Bidomain model on an ellipsoidal domain during the time interval [0 100] ms. We study the time evolution of CPU times and iterations of the two main computational kernels of our electro-mechanical model: the non-linear mechanical solver (NK-BDDC) and linear Bidomain solver (PCG - MAS(3) for the elliptic solve and PCG-BJ for the parabolic solve).

**Figure 7:**
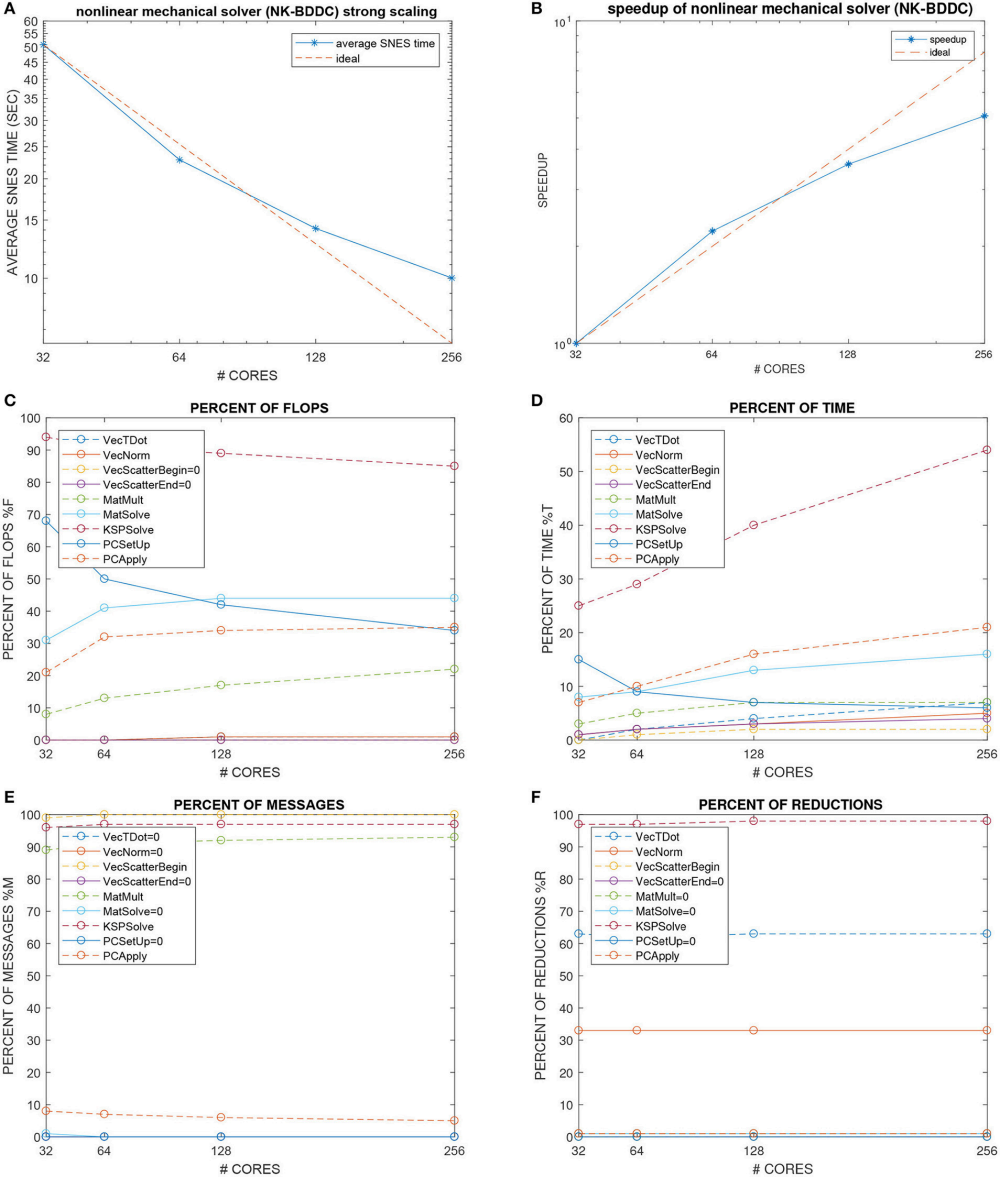
Test 4: strong scalability from 32 to 256 processors of Marconi-A2 of the nonlinear mechanical solver (NK-BDDC). **(A)** Average times and **(B)** associated speedup over the [0 100] ms interval of the nonlinear SNES solver. **(C–F)** Percent summary of flops **(C)**, time **(D)**, messages **(E)**, reductions **(F)**, of the nine main PETSc functions (from VecTDot to PCApply) called by the elliptic solver.

The global mesh size is fixed to 384 × 192 × 48 finite elements while the number of processors is increased from 32 = 8 × 4 × 1 (with local mesh 48 × 48 × 48) to 256 = 16 × 8 × 2 (with local mesh 24 × 24 × 24). Figure 6A shows the timings of the NK-BDDC solver: after an initial superlinear speedup from 32 to 64 cores, the timings still reduce when going to 128 and 256 cores but with worse speedups (see also Figure 7A) and start to increase at 512 cores or more (not shown). Figure 7B shows the number of Newton iterations for each NK-BDDC solve, which remain constant at 4 iterations independently of the number of processors. Figure 7C reports the cumulative GMRES iterations for each NK-BDDC mechanical solve, which increase in time since the Jacobian mechanical system becomes increasingly ill-conditioned due to the spreading of the electrical activation front and subsequent mechanical contraction. The number of iterations is reduced when going from 64 to 128 and to 256 cores, but unexpectedly in the 32 core test we got the lowest iteration counts after 20 ms. Figure 7D shows that the number of PCG iterations for each Bidomain elliptic solve are almost constant independently of the number of processors used. The timings of each Bidomain elliptic (Figure 7E) and parabolic (Figure 7F) solve show a reduction when the number of processors is increased, but with reduced speedup when using 256 cores or more.

As before, we now study in Figure 7 the percent summary (given by the LogView PETSc subroutine) of the main PETSc functions in this strong scaling test for the electro-mechanical solver. We report the percent of: flops (Figure 7C), CPU time (Figure 7D), messages (Figure 7E), reductions (Figure 7F). Again we find quite flat plots, except the time percentages (Figure 7D), where the the KSPSolve percentage grows considerably due mostly to the growth of PCApply and MatSolve, which again we attribute mostly to the nonscalable direct coarse solve (Mumps) of the BDDC preconditioner called by the non-linear SNES solver. The percentage of time (Figure 7D), flops (Figure 7C) and reductions (Figure 7F) are dominated by the PCG solver (KSPSolve), but in Figure 7C the percent of flops of KSPSolve and PCSetUp decreases when the processor count increases, while the percentages of MatSolve, PCApply and MatMult increase. The percentage of messages (Figure 7E) are dominated by vector scattering (VecScatterBegin), linear solves (KSPSolve) and matrix multiplications (MatMult).

## 5. Discussion

We have developed a high-performance parallel solver for cardiac electro-mechanical 3D simulations. After numerical discretization in space with *Q*_1_ finite elements and IMEX operator splitting finite differences in time, the main computational kernels at each time step require: (a) the solution of a non-linear system deriving from the discretization of the cardiac mechanical problem (1) by a parallel Newton-Krylov-BDDC (NK-BDDC) solver, where the Krylov method chosen is GMRES; (b) the solution of the two linear systems deriving from the discretization of the elliptic and parabolic equations in the Bidomain model (Equation 6) by a parallel PCG method with Multilevel Additive Schwarz and Block-Jacobi preconditioners, respectively. The parallelization of our solver has been based on simulations have been performed on the parallel library PETSc (Balay et al., 2012) from the Argonne National Laboratory and large-scale 3D simulations have been run on high-performance supercomputers.

We have investigated the performance of the parallel electro-mechanical solver in both physiological excitation-contraction cardiac dynamics and pathological situations characterized by re-entrant waves.

### 5.1. Bidomain solver

The results have shown that the electrical Bidomain solver is scalable, in terms of both weak and strong scaling, and is robust with respect to the deformation induced by the mechanical contraction. Bidomain weak scaling tests have been performed both on the Mira BG/Q and Marconi-A2 clusters. The two architectures and the number of cores used are different, although the load per core is the same. Thus, we can not compare fairly the performances obtained on the two architectures. However, the CPU times reported in Figure 4A, bottom and Figure 5A have the same order of magnitude, showing that the solution of the Bodomain linear systems on the two architectures exhibit comparable costs.

### 5.2. Mechanical solver

The results have shown that also the mechanical NK-BDDC solver is scalable in terms of non-linear and linear iterations counts, but the CPU timings, especially in the weak scaling test, do not present a scalable behavior. Our results seem to indicate that this increase of CPU timings can be attributed to the increase of computational costs required by the BDDC coarse solver. A possible remedy would be to employ a multilevel BDDC solver, where the coarse problem is solved recursively by a BDDC method with additional local and coarse problems, or to employ an adaptive selection of BDDC primal constraints. The nonscalability and ill-conditioning of the nonlinear mechanical system could also be associated with: (a) the penalty formulation employed to enforce the almost incompressibility of the cardiac tissue; (b) the presence of the stress induced by the active tension contraction model; (c) the particular mechanical boundary condition enforcing zero displacements on a fixed endocardial basal ring and fixed intracavitary endocardial pressure.

### 5.3. Comparison with previous studies

So far, only few studies have developed and investigated parallel numerical solvers for cardiac electro-mechanics. Lafortune et al. (2012) have proposed a fully explicit Monodomain-mechanical solver, obtaining good strong scalability results up to 500 cores. The advantage of our approach with respect to that presented in Lafortune et al. (2012) is that our solver, resulting from a semi-implicit time discretization of the electro-mechanical model, allows larger time step sizes and time adaptivity. Augustin et al. (2016) have developed a very effective electro-mechanical solver, tested on highly accurate patient-specific geometric models and based on Algebraic Multigrid (AMG) preconditioners for both the Bidomain and mechanical systems. The strong scalability results they have reported show a very good performance of AMG applied to the non-linear mechanical system, whereas the AMG preconditioner is less effective for the Bidomain linear system. The advantage of our solver compared to that introduced in Augustin et al. (2016) is that both Multilevel Additive Schwarz and BDDC preconditioners should be more robust than AMG when high order finite elements or isogeometric analysis (see e.g., Charawi, 2017) discretizations are employed. On the other hand, while BDDC preconditioners can be easily constructed for unstructured meshes, Multilevel Additive Schwarz methods are more difficult to implement in case of such grids.

### 5.4. Future work

In order to improve our mechanical solver, further studies could consider the following issues: (a) mixed formulations of the mechanical system based on inf-sup stable displacement-pressure discrete spaces; (b) alternative active tension contraction models; (c) alternative mechanical boundary conditions and pressure-volume relationships involving multielement Windkessel models.

## Author contributions

All authors listed have made a substantial, direct and intellectual contribution to the work, and approved it for publication.

### Conflict of interest statement

The authors declare that the research was conducted in the absence of any commercial or financial relationships that could be construed as a potential conflict of interest.
